# Clinical, Sociodemographic, and Psychological Factors Associated with Transition Readiness in Patients with Epilepsy

**DOI:** 10.3390/brainsci14010021

**Published:** 2023-12-24

**Authors:** Mariacarolina Vacca, Mariana Fernandes, Lorenzo Veronese, Andrea Ballesio, Caterina Cerminara, Cinzia Galasso, Luigi Mazzone, Caterina Lombardo, Nicola Biagio Mercuri, Claudio Liguori

**Affiliations:** 1 Department of Psychology, Sapienza University of Rome, 00185 Rome, Italy; mariacarolina.vacca@uniroma1.it (M.V.); andrea.ballesio@uniroma1.it (A.B.); caterina.lombardo@uniroma1.it (C.L.); 2 Department of Systems Medicine, University of Rome Tor Vergata, Montpellier Street 1, 00133 Rome, Italy; fernandes.mlp@gmail.com (M.F.); lorenzoveronese21@gmail.com (L.V.); cinzia.galasso@uniroma2.it (C.G.); luigi.mazzone@uniroma2.it (L.M.); mercurin@med.uniroma2.it (N.B.M.); 3 Child Neurology and Psychiatry Unit, Department of Neurosciences, Policlinico Tor Vergata Hospital, Viale Oxford 81, 00133 Rome, Italy; caterina.cerminarac@uniroma2.it; 4 Epilepsy Center, Neurology Unit, University Hospital of Rome “Tor Vergata”, 00133 Rome, Italy

**Keywords:** transition readiness, epilepsy, psychological, parents

## Abstract

Background: The transition to adult care for patients with epilepsy is a complicated clinical issue associated with adverse outcomes, including non-adherence to treatment, dropout of medical care, and worse prognosis. Moreover, youngsters with epilepsy are notably prone to emotional, psychological, and social difficulties during the transition to adulthood. Transition needs depend on the type of epilepsy and the epileptic syndrome, as well as on the presence of co-morbidities. Having a structured transition program in place is essential to reduce poor health consequences. A key strategy to optimize outcomes involves the use of transition readiness and associated factors assessment to implement the recognition of vulnerability and protective aspects, knowledge, and skills of these patients and their parents. Therefore, this study aims to provide a comprehensive framework of clinical and psychosocial aspects associated with the transition from pediatric to adult medical care of patients with epilepsy. Methods: Measures examining different aspects of transition readiness and associated clinical, socio-demographic, psychological, and emotional factors were administered to 13 patients with epilepsy (M_age_ = 22.92, SD = 6.56) with (n = 6) or without (n = 7) rare diseases, and a respective parent (M_age_ = 56.63, SD = 7.36). Results: patients showed fewer problems in tracking health issues, appointment keeping, and pharmacological adherence as well as low mood symptoms and moderate resiliency. Moreover, they referred to a low quality of sleep. Notably, parents of patients with rare diseases reported a lower quality of sleep as compared to the other group of parents. Conclusions: Increasing awareness around transition readiness is essential to promote self-management skills of patients with epilepsy and their parents. Anticipating the period of transition could be beneficial, especially to prevent problematic sleep patterns and promote independence in health care management. Parents of patients with epilepsy and rare diseases should be monitored for their mental status which can affect patients’ well-being.

## 1. Introduction

Epilepsy is a highly common chronic neurologic disease presenting unpredictable and recurrent seizures due to anomalous electrical activity in different areas of the cerebral cortex, resulting in a neuronal hypersynchrony or insufficient electrical inhibition [1]. The Task Force of the International League Against Epilepsy (ILAE) defined epilepsy as being characterized by one of the following criteria: (1) at least two unprovoked seizures in the past 24 h, (2) one unprovoked seizure and a ≥60% probability of having an additional seizure over the next 10 years, or (3) reporting an epilepsy syndrome [2]. Epilepsy is one of the most common neurological diseases affecting all ages, with a worldwide prevalence rate of 6.38 per 1000 persons [3]. In Europe, the prevalence of epilepsy has been estimated with ranges varying from 3.3–7.8/1000 inhabitants in the general population [4] to 3.4–5.8/1000 in youngsters, with results largely depending on age-specific populations [5].

Consistently, as compared to other lifetime periods, higher prevalence rates have been observed in adolescents (e.g., 14%) and young adults (e.g., 6.3%) when compared to other ages [6]. As a result, researchers’ interest in studying epilepsy’s implications on the quality of life during these transitional ages increased. The World Health Organization Expert Committee conceptualized adolescence as the period between 10 and 19 years, characterized by profound biological changes and culminating in the maturation of complex cognitive and behavioral abilities [7]. This life phase is characterized by the search for independence and the establishment of a growing consolidation of identity and autonomy, with a parallel strengthening of physical and cognitive competence and subsequent decline in relatedness with parents [8]. The development of autonomy consistently continues during emerging adulthood (19–25 years), another developmental age in terms of identity explorations consisting of dramatic changes in life circumstances (e.g., finishing school, entering the workforce, leaving parental home, marriage) [9]. Notwithstanding the expected evolutive enhancement of self-reliance, emerging adults still tend to perceive their family members as the main sources of assistance [10]. This evidence is critical considering that many studies have shown that parents’ support of autonomy is associated with a range of positive outcomes for their offspring (e.g., high academic performance and low levels of depression [11,12]). 

Young people’s need to overcome age-specific transitional challenges to increase autonomy encounters several barriers and lifestyle complications when living with a chronic condition such as epilepsy [13]. This disease can hinder the achievement of key developmental tasks, negatively impacting independence, peer socialization, and self-esteem [13]. Studies have suggested that epilepsy-related aspects such as exposure to a higher number of anti-seizure medications (ASM) [14], greater seizure severity, and comorbidity [4] may be risk factors for poor a quality of life in youngsters. Moreover, a lack of perceived independence and autonomy in managing their medical condition contributes to this risk [15,16]. Besides that, young people with epilepsy also experience a drastic interruption in their continuity of care when they are transferred from the pediatric to the adult healthcare system [17]. In this regard, the literature indicated that 40–50% of adolescents continue to present with epilepsy throughout life [18], and thus require ongoing specialist healthcare delivery into adulthood [19]. During this process, patients could experience mental distress, fear, and apprehension and even stop their medication [20]. Consequently, it is essential to plan the transition of care for youths with epilepsy, conceived as a coordinated process of transfer from a pediatric environment to an adult-centered care setting [20]. The transition to a care process is different from the mere transfer to care as it focuses on the individual experiences and needs of the patient and includes the multisystemic coordination among different health professionals involved in the education of a patient and his/her family [20]. The consequences of poor transition planning can include increases in healthcare expenditures and a decreased quality of life [21]. The American Epilepsy Society [22] recommends introducing transition to patients of around 10–13 years of age; notwithstanding, empirical research on the best practices to assist with youth transition is limited [23]. Transition is an individualized process, and its successful execution depends not only on a patient’s epilepsy and associated condition (e.g., seizure severity and treatment adherence) but also on the individual level of transition readiness [24].

Transition readiness has been defined as “indications that a patient and those in their support system (e.g., parents and providers) can successfully transition from child-centered to adult-oriented healthcare” ([25], p. 12). These indications help clinicians address self-management of health and improve the health-related skills of young patients and their caregivers [24]. Multi-wave assessment of transition readiness can be crucial to evaluate a patient’s ability to transition successfully in terms of disease knowledge, self-management skills, and expectations from healthcare providers [21]. The literature showed that young patients with epilepsy experiencing high transition readiness also reported a high quality of life [26,27,28] and that certain demographic variables (e.g., socio-economic status, race/ethnicity) may be associated with a higher likelihood to successfully utilize transition readiness skills [29]. The level of transition readiness could be influenced by parents’ agreement and support for initiating independent care [30]. Since parents are the principal healthcare agents for their sons affected by epilepsy, the examination of their perceptions of the transition from pediatric to adult healthcare is needed to develop effective and orchestrated plans for healthcare management [31]. A previous study showed that caregivers and patients differently perceive transition readiness, suggesting the importance of evaluating both transition experiences [32]. Some authors suggested that families of children with epilepsy experience high levels of stress during the parental role transition [33]. This negative impact of the transition process on caregivers’ mental adjustment may also depend on the specific epilepsy characteristics reported by their offspring. For instance, high seizure control of children was associated with better parental quality of life [34], suggesting that the severity of epilepsy may influence parental psychological experiences of the disease. A variable of particular interest in this context is sleep [35,36]. It is well known that in children and adolescents, seizures promote disruption of sleep patterns, and affect the quality, quantity, and architecture of sleep [37]. For instance, in a recent meta-analysis, children with epilepsy were shown to sleep, on average, 34 min less than healthy controls across self-report, actigraphy, 24 h video EEG, and polysomnography measures [38]. Interestingly, the quality and duration of sleep may be associated with quality of life and clinical outcomes (e.g., depression) in children and adolescents with epilepsy [39]. To the best of our knowledge, however, whether sleep may be associated with transition readiness in this population remains to be explored. 

Taken together, the literature indicates that transition readiness is associated with a high perceived quality of life in patients and their caregivers, yet little is known about the levels of specific psychological and personal characteristics associated with this vulnerable phase. Therefore, the present study aims to identify sociodemographic, clinical, and psychological factors presented by young patients with epilepsy and epilepsy syndrome, formally transferred to adult care and their parents. 

## 2. Materials and Methods

### 2.1. Participants and Study Design

Patients affected by epilepsy and epilepsy syndrome, accompanied by their parents, participated in the present study. Participants were admitted to the Transition Clinic and transferred from the Child Neurology and Psychiatry Unit to the Neurology Unit of the University Hospital of Rome “Tor Vergata” in the period between October 2022 and May 2023. Patients and their parents completed a series of questionnaires in the presence of pediatric neuropsychiatry and neurology specialists. Patient and parent forms of each questionnaire were presented to the corresponding participant. Patients completed the questionnaires without consultation with their parents. The diagnoses of epilepsy and epilepsy syndrome were made in accordance with the ILAE recommendations [22] (see above). All the procedures were approved by the ethics committee of University Hospital of Rome Tor Vergata (R.S. 191/17-192/17; Eudract 2017-000990-35).

### 2.2. Measures 

Participants completed a socio-demographic form including questions on age, sex, educational level, socioeconomic status, and clinical information regarding their medical issues and psychological comorbidities. Moreover, a series of questionnaires were administered (see below).

*Transition readiness.* The Epilepsy Transition Readiness Assessment Questionnaire (EpiTRAQ) [40] is a 20-item measure assessing transition readiness in patients and their caregivers. The questionnaire assesses five areas of transition: managing medications, appointment keeping, tracking health issues, talking with providers, and managing daily activities. Responses ranged from 1 to 5, with higher scores indicating greater transition readiness. This scale showed an excellent internal reliability (α = 0.953).

*Medical adherence.* The Self-reported Medication Taking Scale (SRMTS) [41] was completed by patients to measure their levels of medication adherence. An adapted version was provided to caregivers. The SRMTS is composed of seven items rated with a 5-point Likert scale from never (=0) to always (4). Scores ranged from 1 to 13, with higher scores indicating less difficulty in medication assumption. The SRMTS showed very good psychometric properties (α = 0.959).

*Quality of life.* The Pediatric Quality of Life Inventory Epilepsy Module (EpiPedsQL) [42] was used to evaluate the health-related quality of life of patients. It consists of 29 items assessing five domains: impact, cognition, sleep, executive functions, and behavior/mood. An adapted version was employed to assess the parental experience of their offspring’s level of quality of life. Respondents recorded their answers using a 4-point scale ranging from 0 (=never) to 4 (=always). The scale was highly reliable (α = 0.905). The Short Form 12 (SF-12) [43] consists of 12 items that assess eight dimensions of health (physical functioning, role-physical, bodily pain, general health, vitality, social functioning, role-emotional, and mental health). Two overall factors were derived from the SF-12, the physical component score and the mental component score. High scores indicate a lower life quality. This scale demonstrated low internal reliability (α = 0.552).

*Depression.* The Patient Health Questionnaire (PHQ-9) [44] consists of nine items that reflect the nine diagnostic criteria for major depression as defined by the DSM-IV. Each item evaluates symptoms occurring over the past 2 weeks and is scored from 0 (absence of symptoms) to 3 (presence of symptoms nearly every day). The total scores ranged from 0 to 27 and higher scores suggest a high risk of presenting with depression (0–4: absence of depression; 5–9: subthreshold depression; 10–14: moderate depression; 15–27: severe depression). The internal reliability was acceptable (α = 0.767).

*Anxiety.* The General Anxiety Disorder (GAD-7) [45] is a screening tool composed of seven items investigating anxiety-related symptoms as experienced during the past two weeks. Responses are rated on a 4-point Likert scale. The total scores ranged from 0 to 21 and higher scores suggest a high risk of presenting anxiety (0–4: absence of anxiety; 5–9: subthreshold anxiety; 10–14: moderate anxiety; 15–21: severe anxiety). The internal reliability was good (α = 0.864).

*Resiliency.* The Resiliency Scale [46] is a 25-item scale that measures the ability to cope with adversity. Respondents rate items on a Likert scale from 0 to 7. Higher scores indicated a higher level of resilience. This scale showed good psychometric properties (α = 0.741).

### 2.3. Statistical Analysis

First, descriptive statistics were computed to characterize the sample in terms of sex, age, epilepsy onset, epilepsy type, seizure frequency, seizure type, and anti-seizure medications (ASMs). The normality of the data was assessed through the Shapiro–Wilk test. Mann–Whitney *U* tests were used to assess the statistical significance of differences in each psychological aspect investigated between parents of patients with epilepsy and parents of patients with epileptic/rare syndromes. The Mann–Whitney *U* test is a rank-based procedure which is more appropriate with ordinal and not-normally distributed data [47].

## 3. Results

### 3.1. Description of the Sample of Patients

#### 3.1.1. Demographic and Clinical Characteristics

A total of 13 patients (61.5% F; M_age_ = 25.92 ± 6.56; range: 20–40) were included in this study. Of these, seven (57.2% F; M_age_ = 21.71 ± 1.70; range: 20–25) were affected by epilepsy, and six patients (66.7% F; M_age_ = 30.9 ± 6.79; range: 24–40) were affected by epilepsy syndrome and rare diseases with epilepsy. In total, 53.8% of patients reported that they live with at least one parent, whereas 46.2% reported that they also live with brothers and/or sisters. Each patient has a total of 13 years of education, and the majority (77.8%) reported a below to middle socio-economic status. As regards clinical characteristics, five patients had an epilepsy syndrome (tuberous sclerosis complex), one patient had a rare disease with generalized epilepsy, and seven patients were affected by generalized epilepsy. The mean age of onset was 9.15 (SD = 7.19), and the etiology of epilepsy was genetic and structural (n = 5), unknown (n =3), genetic (n = 2), and suspected genetic (n = 3). The frequency of seizures was less than one per year for most patients (n = 7), while the remaining showed one episode per year (n = 3), one–two episodes per year (n = 1), one episode per month (n = 1), and multi-daily episodes (n = 1). Patients did not present with seizures at night, as recorded by the parents or by the epilepsy diary (however, no long-term EEG monitoring was performed at the time of the study). Patients were prescribed one (n = 5), two (n = 7), and three (n = 1) ASMs. More specifically, patients were treated with Levetiracetam (LEV) (n = 4), highly purified Cannabidiol and LEV (n = 1), Everolimus (n = 1), Lacosamide and Everolimus (n = 1), Lamotrigine and Topiramate (n = 1), Valproic Acid (VPA) and LEV (n = 1), VPA and Carbamazepine (n = 1), VPA and Clonazepam (n = 1), VPA and Everolimus (n = 1), and Oxcarbazepine and LEV (n = 1). 

Eleven patients presented multiple comorbidities with psychiatric, neurological, and cardiovascular conditions. Table 1 shows the distribution of these aspects in the epilepsy and epilepsy syndrome groups.

#### 3.1.2. Psychological Characteristics

A total of four patients from the rare diseases outpatient clinic were not asked to complete the questionnaires as they presented moderate to severe intellectual disability. Therefore, a total of nine patients completed the questionnaires investigating levels of transition readiness (EpiTRAQ), medical adherence (SRMTS), quality of life (EpiPedsQL), depression (PHQ-9), anxiety (GAD-7), and resiliency (RS). Considering cutoff scores of PHQ-9, two patients (15.4%) reported mild depressive symptoms and one patient (7.7%) reported moderate depression, whereas the majority (n = 6; 66.7%) did not report depressive symptoms. Mild anxiety symptoms assessed through GAD-7 were detected among three patients (23.1%), whereas one patient (7.7%) presented clinically severe anxiety. Other patients (n = 5; 55.6%) did not report any symptoms. Table 2 summarizes the mean scores for each domain examined.

### 3.2. Description of the Sample of Parents

#### 3.2.1. Demographic and Clinical Characteristics

The parents of the patients included in the study were asked to complete questionnaires regarding their children’s transition and some aspects of their quality of life. In total, 12 parents (75% F; M_age_ = 56.6 ± 7.36; range: 46–74) were recruited (one subject was the parent of two patients included in the study). Half of the parents had secondary education, 25% (n = 3) completed middle school, and 16.7% (n = 2) had post-secondary education. More than half (53.84%) were employed, married (55.5%), and the majority (90%) reported a below to middle socio-economic status. 

#### 3.2.2. Psychological Characteristics

A total of 12 parents completed the questionnaires investigating levels of transition readiness (EpiTRAQ), medical adherence (SRMTS), quality of life (EpiPedsQL, SF-12), depression (PHQ-9), and anxiety (GAD-7) (Table 3). Considering cutoff scores of PHQ-9, three parents (23.1%) reported mild depressive symptoms, two parents (15.4%) reported moderate depression, whereas the majority (n = 6; 54.5%) did not report depressive symptoms. Mild and clinically severe anxiety symptoms assessed through GAD-7 were detected in two parents (15.4%), whereas the majority (n = 7, 63.6%) did not report any anxiety symptoms.

#### 3.2.3. Group Differences

Mann–Whitney *U* tests were used to examine differences between each psychological variable among parents of patients with epilepsy and parents of patients with epilepsy syndrome/rare diseases. The results indicated significant differences in the domains EpiPedsQL-Impact (*U* = 39.500, *p* = 0.005), and EpiPedsQL-Sleep (*U* = 39.500, *p* = 0.007). More specifically, higher scores in sleep difficulties and in the impact of epilepsy on quality of life were observed in the group of parents of patients with epilepsy syndrome/rare diseases as compared to those observed in the group of parents of patients with epilepsy. No other significant results were observed for the other domains (*p* > 0.05).

## 4. Discussion

This study aimed to understand the complex healthcare transition process for patients with epilepsy by examining transition readiness, demographic, clinical, and psychological variables in a sample of patients with epilepsy or epilepsy syndrome/rare diseases, and their parents. This topic is of particular relevance considering the lack of a comprehensive understanding of the experiences of youngsters and parents approaching this ongoing process. Results can support the upskilling of health professionals in the delivery of age-appropriate care and educational programs. A recent systematic review highlighted that transitional plans for the transfer from pediatric to adult care are not often available and evidence on the efficacy of transition programs for young people is limited [48]. Notwithstanding, several psychological problems are frequent (e.g., anxiety, depression) [49] and adversely affect the transition process [50]. Transition programs are not adapted to the age-specific needs of patients and there is no consensus on the evaluation of adequate transitions, the impact on patient experience, and population costs [48]. Therefore, the need to perform a standardized evaluation of patient experience to identify key features of the transitional process and the outcomes defining successful transitional care recently emerged [21,48]. Notwithstanding, the recommended age for an effective transition has been identified as between 12 and 14 years old [51]. Notably, in the present study, the mean age of patients who underwent transitions was 25.92 ± 6.56 years, which was later than recommended. This evidence aligns with previous research [52] and confirms that young adult patients with epilepsy tended to remain in the pediatric clinics, suggesting that the discussion of transition before reaching the age of maturity may not be routinely delivered [53]. This gap in the healthcare system could be seen in the present study by the evidence that patients seemed to report lower levels of readiness in all the domains measured by the EpiTRAQ, as compared to those reported by young adult patients with a wide variety of other medical diseases [54]. Indeed, this finding suggested that epilepsy may be a particularly stressful and challenging condition, even more than other illnesses, due to its unpredictable nature [48], and thus encouraged the expansion of transitional readiness models and theories. We are also aware that these results were obtained in Italy, where the guidelines for transition are probably still lacking and further studies should be published for helping patients, parents, and clinicians in this very sensitive time of patients’ lives.

Young people with epilepsy often report psychosocial, emotional, and behavioral difficulties [16] due to the interaction between neurological (e.g., neurotransmitter-based pathogenic mechanisms), sociodemographic (e.g., financial difficulties), psychological (e.g., social stigma), and epilepsy-related aspects (e.g., use of certain antiepileptic drugs) [16]. In our sample, three patients (33.3%) reported mild to moderate depressive symptoms, whereas four patients (44.4%) presented mild to severe anxiety symptoms. Moreover, patients presented higher scores for both depressive and anxiety symptoms as compared to those reported by studies on young adults without epilepsy [55]. 

These findings prioritized the need to anticipate the transition to adult care in order to prevent the deterioration of mental health in youngsters, by appropriately addressing age-specific difficulties of patients with epilepsy. The mental health difficulties experienced by patients during their transition also concerned sleep patterns, as indicated by the higher score on the relative EpiPedsQL sub-domain. The literature regarding developmental trends of sleep during the transition from adolescence to young adulthood has indicated a general decline in sleep duration and efficiency [56]. Sleep problems during these developmental ages are frequent in patients with epilepsy compared to healthy controls [57] and may affect medication compliance and negatively impact autonomy from caregivers [58]. Moreover, research has shown that caregivers of patients with epilepsy exhibit disrupted sleep patterns and sleep deprivation [59], and parental sleep dysfunction is influenced by a child’s seizure severity [60]. In our investigation, parents of patients with epilepsy syndrome/rare diseases reported greater sleep difficulties and epilepsy-related impact on quality of life as compared to parents of patients with epilepsy. This result concurs well with the evidence that the impact of sleep can be specific considering epilepsy in general and specific epilepsy syndromes (e.g., Lennox-Gastaut syndrome, West syndrome, Tuberous Sclerosis Complex) [61]. These findings seemed to indicate that parents of patients with epilepsy syndrome/rare diseases might particularly benefit from interventions of parental sleep. Considering that the seizure frequency of patients with epilepsy syndrome/rare diseases was low compared to that reported in the previous literature, it appeared evident that sleep problems persist also when seizure frequency is reduced by the use of ASMs. Increased comprehension of the association between poor sleep and epilepsy, possibly also mediated by other factors, may suggest the need to set strategies to improve the sleep quality of patients with epilepsy and their parents to help also mitigate psychiatric comorbidities and behavioral problems at home and school [62].

The present study clearly had some limitations. First, its cross-sectional nature prevents drawing conclusions about the causality of associations. Additional research is needed to explore the associations found with longitudinal methods. Second, the mere use of questionnaires to collect data may be subject to social desirability and recall bias. Future studies should employ more rigorous methods of evaluation, such as qualitative structured interviews or daily diaries to collect subjective data on all the psychological aspects investigated. Moreover, objective measures such as actigraphy or polysomnography to describe sleep parameters are encouraged in the future. Another limitation pertains to the small sample size of the group of patients and parents included, which affected the conclusions of the study in terms of their generalizability. Moreover, the non-random sampling affected our findings in terms of their external validity. Future studies should employ alternative approaches to analyze preference data, such as cluster analysis [63]. The use of non-parametric methods of estimating group differences may have affected our results. Future studies are required to use more sophisticated methods of analysis. For instance, actor–partner interdependence models (APIMs) [64] could be useful in detecting mutual influences of psychological aspects between parents and youths with epilepsy during transition. Finally, future research should collect data on the life history of patients with epilepsy, in order to assess whether deteriorations in sleep patterns may depend on the environment and life events, as mentioned elsewhere [65].

Despite these weaknesses, this study suggests that epilepsy transition programs need to be implemented to ensure the efficient transition of youngsters with epilepsy from pediatric to adult care. One of the main findings of the present study is the delay in transition documented, which reflects the need for guidelines for the transition from child to adult neurological centers in epilepsy care. Another potential practical implication that could be highlighted is the continuous monitoring of mental health in persons with epilepsy approaching adult healthcare, especially given the documentation of depressive and anxiety symptoms found in our sample. In this regard, depression and anxiety were directly related to higher seizure frequency [66]. Therefore, clinicians should take the time to screen these patients in order to combine seizure treatment with mental health interventions through individualized programs. Moreover, starting the introduction of transition planning early could be crucial for improving transition-related emotional problems. Results on sleep further emphasized the need to include assessments of sleep in the management protocols of youngsters with epilepsy. Practitioners are encouraged to promote knowledge of sleep among both patients and their caregivers, especially considering the well-known association between deteriorated sleep and seizure severity [62]. In conclusion, the evidence that emerged indicated the need to improve care not only of patients with epileptic syndrome and epilepsy in comorbidity with rare diseases, but also of their parents, who experience disease-related stress for many years and, considering their increasing age, should be assisted by health professionals who can limit their assistance.

## Figures and Tables

**Table 1:** Clinical characteristics of patients with epilepsy (n = 7) and patients with epilepsy syndrome or epilepsy in comorbidity with rare diseases (n = 6).

Variable	Patients with Epilepsy n (%)	Patients with Epilepsy Syndrome/Rare Diseases n (%)
Epilepsy type		
Generalized	7 (100%)	1 (16.7%)
Focal		4(66.6%)
Focal/generalized		
Epilepsy Syndrome		1 (16.7%)
Seizure type		
Generalized	7 (100%)	2 (33.3%)
Focal		2 (33.3%)
Combined		2 (33.3%)
Aetiology		
Genetic	5 (71.4%)	1 (16.7%)
Unknown	2 (28.6%)	
Combined genetic and structural		5 (83.3%)
Age at epilepsy onset	M = 11.4 (±5.02)	M = 6.4 (±8.89)
Seizure frequency Seizure type: generalized		
<1/year	4 (57.1%)	1 (16.7%)
≥1/year	3 (42.8%)	
Seizure type: focal		
<1/year		2 (33.3%)
≥1/year		2 (33.3%)
Seizure type: combined		
<1/year		1 (16.7%)
≥1/year		
Concomitant ASMs		
1 (%)	4 (57.1%)	1 (16.7%)
2 (%)	3 (42.8%)	4 (66.66%)
≥3 (%)		1 (16.7%)

**Table 2:** Psychological characteristics of patients.

Questionnaires Completed by Patients (n = 9)	Mean	SD	Min	Max
Managing medication (EpiTRAQ)	3.19	0.60	2	4
Appointments keeping (EpiTRAQ)	2.58	0.86	1	4
Tracking health issues (EpiTRAQ)	2.72	1.19	0	4
Tracking with providers (EpiTRAQ)	3.39	0.70	2	4
Daily activities (EpiTRAQ)	2.94	0.82	1	4
Medical adherence (SRMTS)	4.11	4.08	0	13
Cognitive (EpiPedsQL)	0.80	0.82	0	2
Sleep (EpiPedsQL)	0.92	0.40	0	1
Executive function (EpiPedsQL)	4.44	5.22	0	1
Mood (EpiPedsQL)	1.06	0.81	0	2
Depression (PHQ-9)	4.44	2.83	1	10
Anxiety (GAD-7)	5.22	3.42	1	10
Resiliency (RS)	58.89	5.88	51	68
Mental component (SF-12)	48.35		43	59
Physical component (SF-12)	52.68		35	58

**Table 3:** Psychological characteristics of parents.

Questionnaires Completed by Parents (n = 12)	Mean	SD	Min	Max
Medication (EpiTRAQ)	2.91	0.99	1	4
Appointments (EpiTRAQ)	2.65	1.18	1	4
Tracking (EpiTRAQ)	2.79	1.15	1	4
Providers (EpiTRAQ)	3.38	1.13	1	4
Daily activities (EpiTRAQ)	2.90	1.05	0	4
Medical adherence (SRMTS)	7	9.81	0	28
Impact (EpiPedsQL)	3.56	0.78	0	3
Cognitive (EpiPedsQL)	1.31	1.42	0	4
Sleep (EpiPedsQL)	1.36	0.99	0	4
Depression (PHQ-9)	4.55	3.86	0	10
Anxiety (GAD-7)	4.73	4.38	1	13

## Data Availability

The data presented in this study are available on request from the corresponding author. The data are not publicly available due to privacy and ethical restrictions.

## References

[1] Yuen A.W.C., Keezer M.R., Sander J.W. (2018). Epilepsy Is a Neurological and a Systemic Disorder. Epilepsy Behav..

[2] Fisher R.S., Acevedo C., Arzimanoglou A., Bogacz A., Cross J.H., Elger C.E., Engel J., Forsgren L., French J.A., Glynn M. (2014). ILAE Official Report: A Practical Clinical Definition of Epilepsy. Epilepsia.

[3] Fiest K.M., Sauro K.M., Wiebe S., Patten S.B., Kwon C.-S., Dykeman J., Pringsheim T., Lorenzetti D.L., Jetté N. (2017). Prevalence and Incidence of Epilepsy: A Systematic Review and Meta-Analysis of International Studies. Neurology.

[4] Ferro M.A. (2014). Risk Factors for Health-related Quality of Life in Children with Epilepsy: A Meta-analysis. Epilepsia.

[5] Behr C., Goltzene M.A., Kosmalski G., Hirsch E., Ryvlin P. (2016). Epidemiology of Epilepsy. Rev. Neurol..

[6] Banerjee P.N., Filippi D., Allen Hauser W. (2009). The Descriptive Epidemiology of Epilepsy—A Review. Epilepsy Res..

[7] World Health Organization (2015). Adolescent Health Research Priorities: Report of a Technical Consultation.

[8] Inguglia C., Ingoglia S., Liga F., Lo Coco A., Lo Cricchio M.G. (2015). Autonomy and Relatedness in Adolescence and Emerging Adulthood: Relationships with Parental Support and Psychological Distress. J. Adult Dev..

[9] Arnett J.J. (2011). Debating Emerging Adulthood: Stage or Process?.

[10] Phinney J.S., Kim-Jo T., Osorio S., Vilhjalmsdottir P. (2005). Autonomy and Relatedness in Adolescent-Parent Disagreements: Ethnic and Developmental Factors. J. Adolesc. Res..

[11] Levesque C., Zuehlke A.N., Stanek L.R., Ryan R.M. (2004). Autonomy and Competence in German and American University Students: A Comparative Study Based on Self-Determination Theory. J. Educ. Psychol..

[12] Ratelle C.F., Larose S., Guay F., Senécal C. (2005). Perceptions of Parental Involvement and Support as Predictors of College Students’ Persistence in a Science Curriculum. J. Fam. Psychol..

[13] Chew J., Carpenter J., Haase A.M. (2019). Living with Epilepsy in Adolescence—A Qualitative Study of Young People’s Experiences in Singapore: Peer Socialization, Autonomy, and Self-esteem. Child.

[14] Nabavi Nouri M., Puka K., Palmer K., Speechley K.N. (2022). Impact of Number of Anti-Seizure Medications on Long-Term Health-Related Quality of Life in Children with Epilepsy: A Prospective Cohort Study. Seizure Eur. J. Epilepsy.

[15] Baker G.A. (2006). Depression and Suicide in Adolescents with Epilepsy. Neurology.

[16] Stefanidou M., Greenlaw C., Douglass L.M. (2020). Mental Health Issues in Transition-Age Adolescents and Young Adults with Epilepsy. Semin. Pediatr. Neurol..

[17] Geerlings R.P.J., Aldenkamp A.P., Gottmer-Welschen L.M.C., De With P.H.N., Zinger S., Van Staa A.L., De Louw A.J.A. (2016). Evaluation of a Multidisciplinary Epilepsy Transition Clinic for Adolescents. Eur. J. Paediatr. Neurol..

[18] Camfield C.S., Camfield P.R. (2007). Long-term Social Outcomes for Children with Epilepsy. Epilepsia.

[19] Camfield P.R., Camfield C.S. (2014). What Happens to Children with Epilepsy When They Become Adults? Some Facts and Opinions. Pediatr. Neurol..

[20] Healy S.A., Fantaneanu T.A., Whiting S. (2020). The Importance of Mental Health in Improving Quality of Life in Transition-Aged Patients with Epilepsy. Epilepsy Behav..

[21] Smith A.W., Gutierrez-Colina A.M., Roemisch E., Hater B., Combs A., Shoulberg A.M., Modi A.C. (2021). Modifiable Factors Related to Transition Readiness in Adolescents and Young Adults with Epilepsy. Epilepsy Behav..

[22] Galanopoulou A.S., Buckmaster P.S., Staley K.J., Moshé S.L., Perucca E., Engel J., Löscher W., Noebels J.L., Pitkänen A., Stables J. (2012). Identification of New Epilepsy Treatments: Issues in Preclinical Methodology. Epilepsia.

[23] Camfield P., Camfield C., Pohlmann-Eden B. (2012). Transition from Pediatric to Adult Epilepsy Care: A Difficult Process Marked by Medical and Social Crisis: Transition from Pediatric to Adult Epilepsy Care. Epilepsy Curr..

[24] Kanhere S., Joshi S.M. (2023). Transition of Care in Epilepsy. Indian J. Pediatr..

[25] Schwartz L.A., Hamilton J.L., Brumley L.D., Barakat L.P., Deatrick J.A., Szalda D.E., Bevans K.B., Tucker C.A., Daniel L.C., Butler E. (2017). Development and Content Validation of the Transition Readiness Inventory Item Pool for Adolescent and Young Adult Survivors of Childhood Cancer. J. Pediatr. Psychol..

[26] Van Staa A., Van Der Stege H.A., Jedeloo S., Moll H.A., Hilberink S.R. (2011). Readiness to Transfer to Adult Care of Adolescents with Chronic Conditions: Exploration of Associated Factors. J. Adolesc. Health.

[27] Uzark K., Afton K., Yu S., Lowery R., Smith C., Norris M.D. (2019). Transition Readiness in Adolescents and Young Adults with Heart Disease: Can We Improve Quality of Life?. J. Pediatr..

[28] Javalkar K., Johnson M., Kshirsagar A.V., Ocegueda S., Detwiler R.K., Ferris M. (2016). Ecological Factors Predict Transition Readiness/Self-Management in Youth with Chronic Conditions. J. Adolesc. Health.

[29] Traino K.A., Sharkey C.M., Perez M.N., Bakula D.M., Roberts C.M., Chaney J.M., Mullins L.L. (2021). Health Care Utilization, Transition Readiness, and Quality of Life: A Latent Class Analysis. J. Pediatr. Psychol..

[30] Geerlings R.P.J., Gottmer-Welschen L.M.C., Machielse J.E.M., De Louw A.J.A., Aldenkamp A.P. (2019). Failed Transition to Independence in Young Adults with Epilepsy: The Role of Loneliness. Seizure.

[31] Riechmann J., Willems L.M., Boor R., Kieslich M., Knake S., Langner C., Neubauer B.A., Oberman B., Philippi H., Reese J.P. (2019). Quality of Life and Correlating Factors in Children, Adolescents with Epilepsy, and Their Caregivers: A Cross-Sectional Multicenter Study from Germany. Seizure.

[32] Nurre E.R., Smith A.W., Rodriguez M.G., Modi A.C. (2020). Patient, Caregiver, and Provider Perceptions of Transition Readiness and Therapeutic Alliance during Transition from Pediatric to Adult Care in Epilepsy. J. Pediatr. Epilepsy.

[33] Mu P.-F. (2008). Transition Experience of Parents Caring of Children with Epilepsy: A Phenomenological Study. Int. J. Nurs. Stud..

[34] Puka K., Ferro M.A., Anderson K.K., Speechley K.N. (2018). Health-Related Quality of Life in Mothers of Children with Epilepsy: 10 Years after Diagnosis. Qual. Life Res..

[35] Calvello C., Fernandes M., Lupo C., Maramieri E., Placidi F., Izzi F., Castelli A., Pagano A., Mercuri N.B., Liguori C. (2023). Sleep architecture in drug-naïve adult patients with epilepsy: Comparison between focal and generalized epilepsy. Epilepsia Open.

[36] Liguori C., Spanetta M., Fernandes M., Izzi F., Placidi F., Mercuri N.B. (2022). More than sleep and wake disturbances: An actigraphic study showing the sleep-wake pattern dysregulation in epilepsy. Seizure.

[37] Kothare S.V., Kaleyias J. (2010). Sleep and Epilepsy in Children and Adolescents. Sleep Med..

[38] Winsor A.A., Richards C., Bissell S., Seri S., Liew A., Bagshaw A.P. (2021). Sleep Disruption in Children and Adolescents with Epilepsy: A Systematic Review and Meta-Analysis. Sleep Med. Rev..

[39] Hansen B.H., Alfstad K.Å., Van Roy B., Henning O., Lossius M.I. (2016). Sleep Problems in Children and Adolescents with Epilepsy: Associations with Psychiatric Comorbidity. Epilepsy Behav..

[40] Clark S.J., Beimer N.J., Gebremariam A., Fletcher L.L., Patel A.D., Carbone L., Guyot J.A., Joshi S.M. (2020). Validation of EpiTRAQ, a Transition Readiness Assessment Tool for Adolescents and Young Adults with Epilepsy. Epilepsia Open.

[41] Morisky D.E., Green L.W., Levine D.M. (1986). Concurrent and Predictive Validity of a Self-Reported Measure of Medication Adherence. Med. Care.

[42] Modi A.C., Junger K.F., Mara C.A., Kellermann T., Barrett L., Wagner J., Mucci G.A., Bailey L., Almane D., Guilfoyle S.M. (2017). Validation of the Peds QL Epilepsy Module: A Pediatric Epilepsy-specific Health-related Quality of Life Measure. Epilepsia.

[43] Kodraliu G., Mosconi P., Groth N., Carmosino G., Perilli A., Gianicolo E.A., Rossi C., Apolone G. (2001). Subjective Health Status Assessment: Evaluation of the Italian Version of the SF-12 Health Survey. Results from the MiOS Project. J. Epidemiol. Biostat..

[44] Apolone G., Mosconi P. (1998). The Italian SF-36 Health Survey. J. Clin. Epidemiol..

[45] Seo J.-G., Cho Y.W., Lee S.-J., Lee J.-J., Kim J.-E., Moon H.-J., Park S.-P. (2014). Validation of the Generalized Anxiety Disorder-7 in People with Epilepsy: A MEPSY Study. Epilepsy Behav..

[46] Gürgan U. (2003). Resiliency Scale (RS): Scale Development, Reliability and Validity Study. Ank. Univ. Egit. Bilim. Fak. Derg..

[47] Rochon J., Gondan M., Kieser M. (2012). To test or not to test: Preliminary assessment of normality when comparing two independent samples. BMC Med. Res. Methodol..

[48] Goselink R.J.M., Olsson I., Malmgren K., Reilly C. (2022). Transition to Adult Care in Epilepsy: A Systematic Review. Seizure Eur. J. Epilepsy.

[49] Gray V., Palmer L., Whelby K., Vinten J., Gait L. (2017). Exploring the Role of Knowledge of Condition and Psycho-Social Profiles of Young People with Epilepsy during Transition. Epilepsy Behav..

[50] Kwack D.W., Lee H., Lee R., Kim D.W. (2022). Treatment Outcome Following the Transition to Adult Epilepsy Care in Childhood-Onset Epilepsy. Seizure.

[51] Willis L.D. (2020). Transition From Pediatric to Adult Care for Young Adults with Chronic Respiratory Disease. Respir. Care.

[52] Jung S.Y., Yu S.W., Lee K.S., Yi Y.Y., Kang J.W. (2022). Transition from Pediatric to Adult Care among Patients with Epilepsy: Cross-sectional Surveys of Experts and Patients in Korea. Epilepsia Open.

[53] Baca C.M., Barry F., Berg A.T. (2018). The Epilepsy Transition Care Gap in Young Adults with Childhood-Onset Epilepsy. Epilepsy Behav..

[54] Wood D.L., Sawicki G.S., Miller M.D., Smotherman C., Lukens-Bull K., Livingood W.C., Ferris M., Kraemer D.F. (2014). The Transition Readiness Assessment Questionnaire (TRAQ): Its factor structure, reliability, and validity. Acad. Pediatr..

[55] Khubchandani J., Brey R., Kotecki J., Kleinfelder J., Anderson J. (2016). The psychometric properties of PHQ-4 depression and anxiety screening scale among college students. Arch. Psychiatr. Nurs..

[56] Park H., Chiang J.J., Irwin M.R., Bower J.E., McCreath H., Fuligni A.J. (2019). Developmental Trends in Sleep during Adolescents’ Transition to Young Adulthood. Sleep Med..

[57] Zambrelli E., Turner K., Vignoli A., La Briola F., Dionisio S., Malanchini S., Galli F., Canevini M.P. (2020). Sleep Disturbances in Italian Children and Adolescents with Epilepsy: A Questionnaire Study. Epilepsy Behav..

[58] Camfield P., Camfield C., Busiah K., Cohen D., Pack A., Nabbout R. (2017). The Transition from Pediatric to Adult Care for Youth with Epilepsy: Basic Biological, Sociological, and Psychological Issues. Epilepsy Behav..

[59] Larson A.M., Ryther R.C.C., Jennesson M., Geffrey A.L., Bruno P.L., Anagnos C.J., Shoeb A.H., Thibert R.L., Thiele E.A. (2012). Impact of Pediatric Epilepsy on Sleep Patterns and Behaviors in Children and Parents. Epilepsia.

[60] Shaki D., Goldbart A., Daniel S., Fraser D., Shorer Z. (2011). Pediatric Epilepsy and Parental Sleep Quality. J. Clin. Sleep Med..

[61] Carreño M., Fernández S. (2016). Sleep-Related Epilepsy. Curr. Treat. Options Neurol..

[62] Dokurel Çetin İ., Şentürk B., Köse S., Aktan G., Tekgül H., Kanmaz S., Serin M., Yılmaz S., Gökben S. (2023). Sleep Problems in Adolescents with Epilepsy and Their Caregivers: Associations with Behavioural Difficulties. Turk. J. Pediatr..

[63] Kaltoft M.K., Turner R., Cunich M., Salkeld G., Nielsen J.B., Dowie J. (2015). Addressing preference heterogeneity in public health policy by combining Cluster Analysis and Multi-Criteria Decision Analysis: Proof of Method. Health Econ. Rev..

[64] Gonzalez R., Griffin D., Cooper H., Camic P.M., Long D.L., Panter A.T., Rindskopf D., Sher K.J. (2012). Dyadic Data Analysis. APA Handbook of Research Methods in Psychology.

[65] Blanken T.F., Benjamins J.S., Borsboom D., Vermunt J.K., Paquola C., Ramautar J., Dekker K., Stoffers D., Wassing R., Wei Y. (2019). Insomnia disorder subtypes derived from life history and traits of affect and personality. Lancet Psychiatry.

[66] Jackson M.J., Turkington D. (2005). Turkington Depression and anxiety in epilepsy. J. Neurol. Neurosurg. Psychiatry.

